# First Identification of *BoLA-DRB3* Alleles Associated with Differential Susceptibility to Bovine Leukemia Virus Infection and Proviral Load in Chinese Holstein Cattle

**DOI:** 10.3390/pathogens15010034

**Published:** 2025-12-25

**Authors:** Jingyuan Wang, Ryosuke Matsuura, Sonoko Watanuki, Aronggaowa Bao, Noriko Fukushi, Yasunobu Matsumoto, Lin Dong, Guangjun Guo, Chunyang Yao, Changjiang Wang, Feng Wei, Jishan Liu, Xuebo Wang, Fengrong Tian, Jinliang Wang, Yoko Aida

**Affiliations:** 1 Laboratory of Global Infectious Diseases Control Science, Graduate School of Agricultural and Life Sciences, The University of Tokyo, 1-1-1 Yayoi, Bunkyo-ku, Tokyo 113-8657, Japan; jywang0518@g.ecc.u-tokyo.ac.jp (J.W.); matsuura-ryosuke@g.ecc.u-tokyo.ac.jp (R.M.); bao-aronggaowa607@g.ecc.u-tokyo.ac.jp (A.B.); fukushi-noriko@g.ecc.u-tokyo.ac.jp (N.F.); aymat@g.ecc.u-tokyo.ac.jp (Y.M.); 2 Laboratory of Global Animal Resource Science, Graduate School of Agricultural and Life Sciences, The University of Tokyo, 1-1-1 Yayoi, Bunkyo-ku, Tokyo 113-8657, Japan; 3 Shandong Binzhou Animal Science and Veterinary Medicine Academy, Binzhou 256600, China; xiaolinzi213@163.com (L.D.); ggj_122@163.com (G.G.); chunyang_yao@163.com (C.Y.); sd3498@163.com (C.W.); xuehan2008@126.com (F.W.); jishanl2006@126.com (J.L.); 4 Shandong Lvdu Biological Technology Co., Ltd., Binzhou 256600, China; wangxuebo1@outlook.com

**Keywords:** *BoLA-DRB3*, bovine leukemia virus, proviral load, BLV infection, susceptibility, resistance, Chinese Holstein cattle, polymorphism

## Abstract

Bovine leukemia virus (BLV), the most prevalent neoplastic disease of cattle worldwide, is the causative agent of enzootic bovine leukosis. Polymorphisms in the bovine leukocyte antigen (*BoLA*)*-DRB3* gene can influence host immune responses to pathogens, including BLV. However, the associations between specific *BoLA-DRB3* alleles, BLV proviral load (PVL), a useful index for estimating disease progression and transmission risk, and BLV infection in Chinese cattle remain unknown. In this study, we identified 28 previously reported alleles in 289 Holstein cattle from Shandong Province, China, using polymerase chain reaction sequence-based typing. We further investigated whether *BoLA-DRB3* polymorphisms influenced infection status and identified BoLA-DRB3*011:01 as an allele associated with susceptibility to BLV infection. An association analysis of allele frequencies between cattle with high and low PVL demonstrated that BoLA-DRB3*014:01:01 was significantly associated with low PVL. Farms with a higher frequency of cattle carrying BoLA-DRB3*014:01:01 had lower mean PVL values than farms with a lower frequency, indicating that resistant alleles are linked to low PVL levels. To our knowledge, this is the first study to demonstrate that *BoLA-DRB3* polymorphisms associate with differential susceptibility to BLV infection and PVL in Holstein cattle in China. These findings may contribute to BLV control and eradication efforts through genetic selection.

## 1. Introduction

The major histocompatibility complex (MHC) plays a central role in the adaptive immune response in all vertebrate animals [1]. The discovery of the cattle MHC has been attributed to Amorena and Stone [2], and Spooner et al. [3]. Genetic regions identified using serological reagents produced by skin transplantation and alloimmunization were named the bovine leukocyte antigen (*BoLA*) system [4]. The *BoLA* region on chromosome 23 is organized similarly to the human MHC, although notable differences exist, including the division of class II loci into two regions, designated IIa and IIb [5]. Among the *BoLA* class II loci, *BoLA-DRB3* is the most polymorphic, with 387 alleles registered in the Immuno Polymorphism Database (IPD)-MHC database (https://www.ebi.ac.uk/ipd/mhc/group/BoLA/, accessed on 24 November 2025) [6], many of which are associated with various diseases. In infectious diseases, *BoLA-DRB3* has been associated with mastitis [7,8,9], tick-borne diseases [10,11], foot-and-mouth disease [12,13], bovine herpesvirus 1 [14], bovine papillomavirus-induced bladder cancer [15], neosporosis [16], *Staphylococcus* spp. [8], and *Escherichia coli* [8], but not with susceptibility to bovine tuberculosis [17]. Moreover, *BoLA-DRB3* polymorphisms influence dairy productivity traits, such as milk quality and production during mastitis infection [18], the microbiota of colostrum and milk [19], and reproduction rates in neosporosis [16]. Notably, *BoLA-DRB3* is consistently associated with mastitis and somatic cell count [9,20,21,22,23,24].

The association between *BoLA-DRB3* and bovine leukemia virus (BLV) has been one of the most intensively studied topics. BLV, a retrovirus closely related to human T-cell leukemia virus type 1 (HTLV-1), causes enzootic bovine leukosis (EBL), the most common neoplastic disease in cattle [25,26,27,28,29,30]. BLV infection results in substantial economic losses, including reduced meat and milk production, shorter productive life [31,32,33,34,35,36], increased premature culling and carcass condemnation [34,37,38,39,40], higher veterinary and management costs linked to immunosuppression [41,42], trade restrictions [27,43,44], and losses associated with the disposal of lymphoma-affected animals [31,45].

As a retrovirus, BLV integrates its genome into host DNA as a provirus, resulting in lifelong infection [25]. Proviral load (PVL) correlates with viral transmission risk and disease outcome and is therefore recognized as a key marker for BLV control and disease monitoring [46,47,48,49,50,51,52,53]. PVL is also influenced by *BoLA-DRB3* polymorphism. Resistant alleles associated with low PVL (LPVL) and susceptible alleles associated with high PVL (HPVL) have been identified in Japanese Black and Holstein cattle [49,54,55,56]. Cattle carrying susceptible alleles show higher infectivity and elevated PVL, placing them at greater risk of horizontal transmission. In contrast, cattle with resistant alleles have lower PVL and reduced infectivity, resulting in a lower transmission risk [47,57,58]. Vertical transmission risk is also markedly reduced in dams and calves with resistant alleles compared with those carrying susceptible alleles [46]. A large-scale field study across four farms in Japan demonstrated that an integrated eradication strategy, using resistant cattle as a biological barrier and preferentially eliminating susceptible cattle, effectively reduced BLV prevalence and PVL, even under group-housing conditions. One farm achieved BLV-free status after 3 years, and another after 6 years [58]. Similar approaches may be effective in free-stall barns, free barns, and grazing systems by gradually increasing the proportion of resistant cattle to reduce contact between BLV-negative animals and animals with HPVL. Thus, eradication strategies guided by resistant and susceptible *BoLA-DRB3* alleles represent a practical tool for controlling BLV infection. However, despite more than 15.2 billion cattle worldwide (https://www.globalnote.jp/post-15229.html, accessed on 24 November 2025), most *BoLA-DRB3* diversity remains uncharacterized.

Holstein cattle were first introduced into China in the mid-19th century by European and North American merchants and missionaries. During this period, Holstein cattle were imported from North America, Russia, and Europe for local breeding or crossbreeding with indigenous Chinese Yellow cattle [59]. As of February 2025, the Chinese dairy herd numbered 6.11 million animals, including 659,000 in Shandong Province [60]. Despite the large and expanding dairy population, the diversity of the *BoLA-DRB3* gene in Chinese Holstein cattle has not yet been fully investigated.

BLV is highly prevalent in most regions worldwide [61], except in several Western European countries that have successfully implemented eradication programs [62,63]. In countries without compulsory control measures, the virus continues to spread because of the absence of effective treatment options. Reported BLV seroprevalence in China ranges from 21.1% to 41.9% [64,65,66]. Therefore, genetic selection may contribute to disease control and eradication efforts in China. Understanding the distribution of *BoLA-DRB3* alleles in Chinese cattle is essential for developing such strategies. However, no analysis of *BoLA-DRB3* has been conducted in China. The objective of this study was to characterize the distribution of *BoLA-DRB3* alleles in Holstein cattle and identify alleles associated with BLV PVL and BLV infection in Shandong Province, China.

## 2. Materials and Methods

### 2.1. Animals, Sampling, DNA Extraction, and Serum Isolation

From 2024 to 2025, whole blood samples were collected from 289 Holstein dairy cattle aged 3–5 years from three dairy farms in Binzhou City, Shandong Province, China. Blood was collected into tubes containing ethylenediaminetetraacetic acid (EDTA). Genomic DNA was extracted using UE Blood Genomic DNA Miniprep Kit (UElandy Corporation, Suzhou, China) according to the manufacturer’s instructions, and DNA concentration was measured using a NanoDrop One Spectrophotometer (Thermo Fisher Scientific, Waltham, MA, USA). DNA samples were adjusted to 30 ng/μL for use in the BLV-CoCoMo Dry Dual qPCR reagent (Dry Dual-CoCoMo assay) [67] to quantify BLV PVL. Plasma was separated by centrifugation for the detection of anti-BLV antibodies.

### 2.2. Ethical Approval

All animals were handled in accordance with the regulations of the Animal Ethics Committee of the Shandong Binzhou Animal Science and Veterinary Medicine Academy (Approval Number: 20240510-01; Approval date: 20 April 2024) and the Animal Experiments Committee of the University of Tokyo (Approval Number: p22-2-030; Approval date: 22 May 2022).

### 2.3. Detection of Anti-BLV gp51 Antibodies

Anti-BLV gp51 antibodies were detected using an anti-BLV antibody enzyme-linked immunosorbent assay (ELISA) Kit (Nippon Gene, Toyama, Japan) following the manufacturer’s instructions.

### 2.4. Quantification of BLV PVL Using the Dry Dual-CoCoMo Assay

BLV PVL was quantified using the Dry Dual-CoCoMo assay and GeneAce Probe qPCR Mix II (Nippon Gene), as previously described [53,67,68]. Briefly, a 183 bp fragment of the BLV long terminal repeat (LTR) region and a 151 bp sequence of the *BoLA-DRA* gene were amplified using CoCoMo-FRW (5′-AATCCMNMYCYKDAGCTGCTGAYYTCACCT-3′ and 5′-ATCCACACCCTGAGCTGCTGCACCTCACCT-3′ mixed with 1:10) and CoCoMo-REV (5′-TTGCCTTACCTGMCSSCTKSCGGATAGCCGA-3′), and DRA-FW (5′-CCCAGAGTATGAAGCTCCAGCCC-3′) and DRA-RW (5′-CCCTCGGCGTTCAACGGTGT-3′) primers pair, respectively. Detection was performed using the degenerate CoCoMo primer mix with a 6-carboxyfluorescein (FAM)-labeled LTR minor groove binder (MGB) probe (5′-FAM-CTCAGCTCTCGGTCC-NFQ-MGB-3′) and the *BoLA-DRA* primer mix with a VIC-labeled DRA MGB probe (5′-FAM-TGTGTGCCCTGGGC-NFQ-MGB-3′). The *BoLA-DRA* gene served as the internal control for normalization of host genomic DNA.

All qPCR reagents for the Dry Dual-CoCoMo assay were freeze-dried in 0.2 mL PCR tubes at Nippon Gene Co., Ltd., and stored at room temperature until use. For each reaction, 15 μL of PCR-grade water and 5 μL of DNA (30 ng/μL) were added to the tubes. After vortexing and brief centrifugation, qPCR was performed using a LightCycler^®^ 480 System II (Roche Diagnostics, Mannheim, Germany) under the following cycling conditions: 95 °C for 10 min, followed by 45 cycles of 95 °C at 15 s and 60 °C for 1 min. PVL was calculated using the following formula:

(BLV LTR copy number/*BoLA-DRA* copy number) × 10^5^.

### 2.5. Determination of BLV Infection

BLV infection status was determined by combining results from the Dry Dual-CoCoMo assay (BLV provirus detection) and ELISA (anti-BLV antibody detection) at the time of blood collection. Serum samples were diluted 1:50 and tested by ELISA according to the manufacturer’s instructions. Optical density values were used to calculate the S/P ratio, with a cut-off of 0.3 to distinguish BLV-positive and BLV-negative samples. For the Dry Dual-CoCoMo assay, cattle were considered BLV-negative (−) if PVL = 0 and BLV-positive (+) if PVL ≥ 1, following previous criteria [48]. Animals that tested positive by either ELISA or qPCR were classified as BLV-positive.

### 2.6. Genotyping of BoLA-DRB3

*BoLA-DRB3* alleles were determined using the PCR sequence-based typing (SBT) method as described previously [69]. Exon 2 of *BoLA-DRB3* was amplified using DRB3-forward (5′-CGCTCCTGTGAYCAGATCTATCC-3′) and DRB3-reverse (5′-CACCCCCGCGCTCACC-3′) primers. PCR products were purified using a Gel Purification Kit (spin-column) (Bioteke Corporation, Beijing, China) and sequenced by General Bio Co., Ltd. (Anhui, China). Alleles were identified with Assign 400ATF software (version 1.0.2.41; Conexio Genomics, Fremantle, Australia).

### 2.7. Statistical Analysis

Allele frequencies were determined by direct counting. Associations between *BoLA-DRB3* alleles and BLV infection status were assessed using Fisher’s exact test by comparing allele frequencies between BLV-positive and BLV-negative cattle. Odds ratios (ORs) were calculated to estimate the strength of association. Similarly, the relationship between *BoLA-DRB3* alleles and BLV PVL categories (LPVL vs. HPVL) was evaluated using Fisher’s exact test. Differences in the frequency of cattle with BoLA-DRB3*014:01:01 among farms were also analyzed using Fisher’s exact test. Statistical power was calculated from posterior probabilities, and a value of ≥0.8 was considered adequate. Statistical analyses were conducted using GraphPad Prism 7 (GraphPad Software Inc., La Jolla, CA, USA). A *p*-value < 0.05 was considered statistically significant and Bonferroni correction was applied for the strict statistical significance threshold at *p*-value < 0.05/number of alleles.

## 3. Results

### 3.1. Genotyping of BoLA-DRB3 of Chinese Holstein Cattle

To identify whether *BoLA-DRB3* alleles are associated with susceptibility or resistance to BLV Infection and PVL in Chinese Holstein cattle, 289 dairy cattle from three BLV-positive farms in Binzhou City, Shandong Province, China, were genotyped for *BoLA-DRB3* using a PCR-SBT assay (Table 1). Twenty-eight previously reported alleles were identified in the 289 cattle. Eight alleles were present at frequencies greater than 2%. These eight most frequent alleles were DRB3*001:01, DRB3*009:02, DRB3*010:01, DRB3*011:01, DRB3*012:01, DRB3*014:01:01, DRB3*015:01, and DRB3*027:03, with frequencies of 32.70%, 2.25%, 5.88%, 8.30%, 2.25%, 17.30%, 21.45%, and 2.08%, respectively. The results showed that DRB3*001:01 was the most abundant allele (32.70%), DRB3*015:01 was the second most frequent (21.45%), and DRB3*014:01:01 was the third most frequent (17.30%). In contrast, the remaining 20 alleles were detected at lower frequencies, ranging from 0.87% to 0.17%.

### 3.2. BLV Prevalence of Chinese Holstein Cattle by Dry Dual-CoCoMo Assay and Serological Tests

To determine BLV infection status in the 289 dairy cattle genotyped for *BoLA-DRB3*, as shown in Table 1, real-time PCR was performed using the Dry Dual-CoCoMo assay targeting the BLV LTR region, which has higher sensitivity than other real-time PCR assays for field samples [48,70]. Serological testing was also performed using ELISA, which is routinely used for BLV diagnosis in Japan (Table 2). Of the examined animals, 28 (31.5%) of 89 cows at H1 Farm, 18 (18.0.%) of 100 cows at H2 Farm, and 8 (8.0.%) of 100 cows at H3 Farm were positive for BLV provirus (Dry Dual-CoCoMo assay) and anti-BLV antibodies (ELISA). Overall, 54 (18.7.%) of the 289 cattle were positive by both methods. Conversely, 61 (68.5%) of 89 cows at H1 Farm, 79 (79.0.%) of 100 cows at H2 Farm, and 90 (90.0.%) of 100 cows at H3 Farm were negative for BLV provirus and anti-BLV antibodies. However, four cows (1.4%) were positive for the BLV provirus but negative for anti-BLV antibodies, and one cow (0.3%) was positive only for anti-BLV antibodies. In this study, animals with at least one positive (qPCR or ELISA) were classified as BLV-positive.

### 3.3. Analysis of the Association Between BoLA-DRB3 and BLV Infection in Chinese Holstein Cattle

To clarify the association between *BoLA-DRB3* alleles and BLV infection, allele frequencies were compared between non-infected (n = 230) and infected (n = 59) cattle (Table 3). BoLA-DRB3*001:01 was the most frequent allele in both groups. Similarly, DRB3*015:01, DRB3*014:01:01, and DRB3*011:01 were the second, third, and fourth most frequent alleles in infected and non-infected cattle. In contrast, DRB3*002:01, DRB3*003:01:01, DRB3*003:02:01, DRB3*004:01, DRB3*006:01, DRB3*007:01, DRB3*008:01, DRB3*009:01, DRB3*009:02, DRB3*016:01, DRB3*027:01, DRB3*031:01, DRB3*032:02, DRB3*044:01, and DRB3*045:01 were present only in the non-infected group. Alleles with OR > 1 were considered candidates for susceptibility, and alleles with OR < 1 as candidates for resistant. Although OR values > 1 were observed for DRB3*001:01, DRB3*005:03, DRB3*011:01, DRB3*011:02, DRB3*015:01, and DRB3*018:01, only DRB3*011:01 showed a significant association (*p* < 0.01), indicating that this allele was associated with susceptibility to BLV infection. No allele showed a significant association with resistance.

### 3.4. Estimation of BLV PVL in Chinese Holstein Cattle and Classification into Three PVL Groups

PVL is an important diagnostic index for determining disease progression and transmission risk in BLV infection. PVL in peripheral blood correlates strongly with infection status, transmission potential, and disease development [51,53,71]. Therefore, PVL was measured in 289 dairy cattle using the Dry Dual-CoCoMo assay (Table 4). PVL values ranged from 0 to 128,378 copies per 10^5^ cells, with a mean of 35,569 copies per 10^5^ cells (Table 4). Following previous studies [72,73], cattle were classified into three groups based on PVL distribution: the top 30% as HPVL, the bottom 30% as LPVL, and the remaining animals as moderate PVL. As a result, the LPVL group included cattle with PVL values of 0 ≤ PVL ≤ 3606 (19 cows), the moderate PVL group included cattle with PVL values of 3720 ≤ PVL ≤ 50,743 (20 cows), and the HPVL group included cattle with PVL values of 51,531 ≤ PVL ≤ 128,378 (20 cows) (Table 5).

### 3.5. Analysis of the Association Between BoLA-DRB3 and BLV PVL in Chinese Holstein Cows

To identify whether *BoLA-DRB3* alleles were associated with susceptibility or resistance to BLV PVL, we calculated the allelic frequencies and compared the frequency distribution of alleles in LPVL and HPVL cows using direct counting and OR and *p*-value calculations based on the chi-square test (Table 6). Alleles with an OR > 1 were categorized as resistant, whereas those with an OR < 1 were considered susceptible. Our results showed that BoLA-DRB3*001:01 (35.0%), DRB3*015:01 (25.0%), and DRB3*011:01 (20.0%) were the most frequent alleles in cattle with HPVL. In the LPVL group, the four most frequent alleles were BoLA-DRB3*001:01 (39.5%), DRB3*014:01:01 (23.7%), DRB3*011:01 (18.4%), and DRB3*15:01 (13.2%). A significant association was observed between BoLA-DRB3*014:01:01 and LPVL (*p* = 0.001; statistical power = 0.86), indicating that DRB3*014:01:01 is a resistant allele. Because this allele was present in the LPVL group but absent in the HPVL group, the OR could not be calculated. In contrast, no *BoLA-DRB3* allele showed a significant association with the HPVL profile.

### 3.6. Comparison of BLV PVL and Frequency of Cattle Carrying the Resistant Allele BoLA-DRB3*014:01:01 in Three Dairy Farms

**Figure 1:**
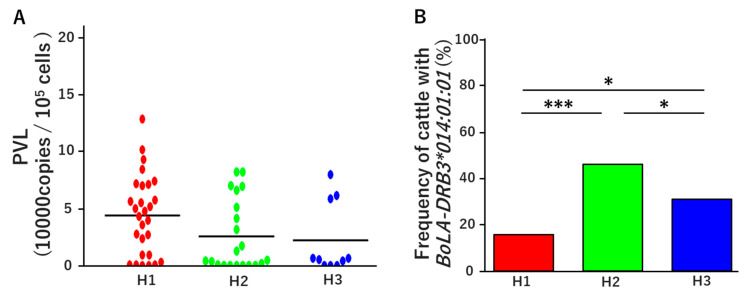
Comparison of proviral loads (PVLs) (**A**) and frequencies of cattle carrying the resistant allele BoLA-DRB3*014:01:01 (**B**) among three dairy farms in China. A total of 289 blood samples were collected from three different farms (H1, H2, and H3) in Binzhou City, Shandong Province, China, and screened for BLV infection using enzyme-linked immunosorbent assay (ELISA) to detect anti-BLV gp51 antibodies and real-time PCR with the Dry Dual-CoCoMo assay. Animals with at least one positive result (ELISA or qPCR) were classified as BLV-positive. (**A**) Comparison of mean PVL across the three farms. The *X*-axis shows farm classification, and the *Y*-axis shows PVL. (**B**) Genomic DNA from all 289 cattle was typed for *BoLA-DRB3* alleles using the PCR-SBT method. The frequency of cattle carrying the resistant allele BoLA-DRB3*014:01:01 was compared among farms, and *p*-values were calculated using Fisher’s exact test. The *X*-axis shows farm classification, and the *Y*-axis shows allele frequency. Asterisks indicate significant differences (* *p* < 0.05 and *** *p* < 0.001).

The BLV PVL was compared among the three farms. At H1 Farm, PVL ranged from 8 to 128,378 copies per 10^5^ cells, with a mean of 43,772 copies per 10^5^ cells. At H2 Farm, PVL ranged from 0 to 82,097 copies per 10^5^ cells with a mean of 25,479 copies per 10^5^ cells. At H3 Farm, PVL ranged from 9 to 79,832 copies per 10^5^ cells, with a mean of 21,988 copies per 10^5^ cells (Table 4 and Figure 1A). We also evaluated the number of cattle carrying the resistant allele BoLA-DRB3*014:01:01 on each farm: 14 cows (15.7%) at H1 Farm, 46 cows (46.0%) at H2 Farm, and 31 cows (31.0%) at H3 Farm (Figure 1B). Our results showed that Farms H2 and H3, which had a higher frequency of cattle carrying BoLA-DRB3*014:01:01, also had lower mean PVL values than Farm H1, which had a lower frequency of this resistant allele.

## 4. Discussion

This study provides information on the distribution of the *BoLA-DRB3* allele and the identification of *BoLA-DRB3* alleles associated with susceptibility or resistance to BLV infection and BLV PVL in Holstein cattle in China. First, we successfully identified 28 previously reported *BoLA-DRB3* alleles in 289 Chinese Holstein cattle using the PCR-SBT method. To the best of our knowledge, this is the first study to report the distribution of *BoLA-DRB3* alleles in Holstein cattle in China. Second, the frequencies of BLV-infected and uninfected cattle within each *BoLA-DRB3* allele were compared. BoLA-DRB3*011:01 was identified as a susceptible allele for BLV infection in the tested Holsteins, whereas no other significant association between *BoLA-DRB3* alleles and BLV infection was found. To our knowledge, this is the first report demonstrating that *BoLA-DRB3* polymorphisms affect BLV infection in Holstein cattle in China. Third, our association analysis of *BoLA-DRB3* alleles in individuals with HPVL and LPVL demonstrated for the first time that DRB3*014:01:01 is a resistant allele in Holstein cattle in China. Fourth, we found that farms with a higher frequency of cattle carrying the resistant allele BoLA-DRB3*014:01:01 had lower mean PVL values than farms with a lower frequency. This indicates that when the proportion of cattle carrying the resistant allele exceeds a certain threshold, PVL levels tend to be lower. This result suggests that the PVL-resistant *BoLA-DRB3* allele is strongly linked to low PVL after BLV infection and is not associated with a high transmission risk. Thus, our results demonstrate that *BoLA-DRB3* polymorphism influences susceptibility to BLV PVL and BLV infection in Holstein cattle in China. These results may be useful for BLV control and eradication through genetic selection.

In this study, samples from 289 Holstein cattle from three farms demonstrated animal- and herd-level BLV positivity rates of 18.7% and 100%, respectively, in Binzhou City, Shandong Province, China. In particular, we concluded that BLV PVL levels are well associated with animal-level prevalence under field conditions in China. For example, 28/89 samples (31.5%) at H1 Farm, 21/100 (21.0%) at H2 Farm, and 10/100 (10.0%) at H3 Farm were positive for BLV infection, with mean PVL values of 43,772 copies per 10^5^ cells, 25,479 copies per 10^5^ cells, and 21,988 copies per 10^5^ cells, respectively. Furthermore, the proportions of cows carrying the resistant allele BoLA-DRB3*014:01:01 were 14 (15.7%) of 89 at H1 Farm, 46 (46.0%) of 100 at H2 Farm, and 31 (31.0%) of 100 at H3 Farm. These results clearly demonstrate that Farms H2 and H3, which had a higher proportion of cattle carrying BoLA-DRB3*014:01:01, had lower mean PVL values and BLV prevalence than Farm H1, which had a lower proportion. This indicates that BLV-infected cattle with resistant alleles are less likely to develop HPVL and are less likely to act as infection sources for BLV-free cattle in Chinese dairy herds, supporting the results of previous studies [47,54,55,57,58,74,75,76,77]. Thus, our results confirm that BLV-infected cattle carrying resistant alleles are at a low risk of BLV transmission because of their LPVL.

Our result demonstrated that DRB3*014:01:01 is a resistant allele for BLV PVL in Chinese Holstein cattle. This resistance finding is based on a count of 0 in the HPVL group, leading to an “infinite” OR. The *p*-value obtained from Fisher’s exact test met the significance level adjusted by the Bonferroni method. However, because this result is highly sensitive to sample size because it relies on the complete absence of the allele in one small subgroup (n = 20), we are currently planning to demonstrate the reproducibility of resistance for BLV PVL of BoLA-DRB3*014:01:01 in Chinese Holstein cattle using a large number of samples.

The kinetic study of BLV infectivity conducted in Japanese Holstein cattle from 2017 to 2019 showed that susceptible cattle exhibited stronger BLV infectivity than both resistant and neutral cattle, and the order of intensity of BLV infectivity ranked as follows: susceptible cattle > neutral cattle > resistant cattle [47]. In addition, BLV infectivity showed a strong positive correlation with PVL at each testing point [47]. Based on these results, it can be inferred that the BoLA-DRB3*014:01:01 alleles rerated with resistance to BLV PVL likely indicate long-term effectiveness rather than an observation at a single time point. By contrast, because long-term observations have not been conducted for *BoLA-DRB3* alleles identified as resistant or susceptible to BLV infection, but not to PVL, it remains unclear non-infected cattle are simply unexposed to BLV or are in an early incubation period. Therefore, it is imperative to investigate the long-term effect of the BoLA-DRB3*011:01 allele, which was identified as conferring susceptibility to BLV infection in the present study.

*BoLA-DRB3* is a highly polymorphic gene, with 387 alleles registered in the IPD-MHC database. Therefore, the diversity and distribution of *BoLA-DRB3* in different geographic regions and breeds are of particular interest to cattle breeders and veterinary geneticists when designing breeding strategies to increase the number of disease-resistant offspring [78]. Documented *BoLA-DRB3* diversity in Holstein cattle from different geographic regions is summarized in Table 7. Although allele diversity varies across locations, the major alleles BoLA-DRB3*001:01, DRB3*011:01, and DRB3*015:01 are commonly observed in Holstein cattle, which supports our results. In this study, 28 previously reported *BoLA-DRB3* alleles were identified in 289 Holstein cattle in Binzhou City, Shandong Province, China, and DRB3*001:01, DRB3*015:01, and DRB3*014:01:01 were the most frequent alleles. Notably, BoLA-DRB3*001:01 and BoLA-DRB3*011:01, as well as BoLA-DRB3*014:01:01, are highly common in Holstein populations in other countries (Table 7). In contrast, different breeds show different major alleles; for example, the most abundant allele in Bolivian Nellore is BoLA-DRB3*028:01 [79], in Sudanese Kenana it is BoLA-DRB3*024:01 [80], and in Japanese Jersey it is BoLA-DRB3*045:01 [81], which is rarely found in Holstein cattle. As of February 2025, the Chinese dairy cow inventory totaled 6.11 million head, including 659,000 in Shandong Province [60]. By the end of 2024, the national beef cattle inventory reached 100.47 million head, with 1.798 million in Shandong Province [82]. However, the allelic diversity of the *BoLA-DRB3* gene in most cattle breeds in China remains largely unclear. Therefore, there is an urgent need to clarify *BoLA-DRB3* allele diversity across different cattle breeds in China.

To determine the effect of *BoLA-DRB3* on BLV infection in Holstein cattle in China, we compared *BoLA-DRB3* allele frequencies between BLV-infected cattle (59 heads) and BLV-non-infected cattle (230 heads). BoLA-DRB3*011:01 was the third most frequent allele present in the infected group and was associated with susceptibility to BLV infection in Chinese Holstein cattle (OR = 2.14; *p* < 0.01). In contrast, in two previous studies, BoLA-DRB3*011:01 was associated with resistance to disease progression to the lymphoma stage in Holstein cattle in Japan and Iran [51,70]. Likewise, one previous study reported that BoLA-DRB3*011:01 was associated with susceptibility to BLV infection in Vietnamese Holstein cattle [72]. In addition, BoLA-DRB3*011:01 was previously reported to be a neutral allele that was not associated with resistance or susceptibility to BLV PVL [72]. Thus, the association between *BoLA-DRB3* and BLV infection profiles should be validated in future studies using a larger number of animals.

BLV PVL, which represents the number of copies of a provirus, is associated with disease progression [53,84,85] and transmission risk [47,51,53,57,58,71,86], suggesting that PVL determination is an important diagnostic marker. It has been reported that BLV-infected cows with HPVLs are at a higher risk of spreading the virus [86,87,88,89] and developing EBL [53,84,85]. Therefore, we attempted to identify *BoLA-DRB3* alleles associated with susceptibility or resistance to BLV PVL. In this study, we found that BoLA-DRB3*014:01:01 (*p* = 0.001) was associated with LPVL in BVL-infected Chinese Holstein cattle. In particular, we confirmed that the PVL-resistant BoLA-DRB3*014:01:01 allele was strongly linked to low PVL, as shown by the correlation between PVL levels and the proportion of cattle carrying this resistant allele. However, previous study reported that the significant factors for the BLV transmission and PVL level were found to be cattle housing conditions, the presence of horseflies in summer, breeding systems, dehorning practices, and colostrum feeding using logistic model with a random herd effect [51,90]. Because the effects of prevalence of BLV, farm management practices, insect control, and biosecurity have not been examined in this study, more detailed analyses are required. On the other hand, our findings are supported by previous studies in which BoLA-DRB3*002:01 and DRB3*014:01:01 were associated with BLV resistance [55,75]. In addition, the BoLA-DRB3*009:02 allele is known to be strongly associated with persistent lymphocytosis resistance and the LPVL profile in Holstein cattle [55,57,76]. Previous PVL association studies have also reported BoLA-DRB3*015:01 and DRB3*012:01 as PVL-related alleles [55]. However, no *BoLA-DRB3* allele showed a significant association with HPVL in the present study. Because BLV PVL is the most variable quantitative index for assessing BLV transmission risk [57,58], information on disease-susceptible and disease-resistant alleles may help eliminate BLV from farms without separating cows into multiple sheds. Therefore, it is important to examine the association between *BoLA-DRB3* and BLV PVL in a larger number of animals. In addition, resistant or susceptible alleles may vary depending on cattle breed, geographic location, and sample collection [78]. Thus, studying the association between *BoLA-DRB3* and BLV in different regions and cattle breeds in China is necessary.

Although BLV infects cattle worldwide, effective treatments and vaccines are currently unavailable. Therefore, breeding strategies based on *BoLA-DRB3* polymorphism are promising for reducing the burden of BLV-induced lymphoma. Recent studies have shown that BLV infectivity in cattle with PVL-resistant *BoLA-DRB3* alleles is lower and that these cattle have reduced horizontal [47,57,58] and vertical [46] transmission potential. This is also reflected in our data, which show that when the proportion of cattle carrying the resistant allele BoLA-DRB3*014:01:01 exceeds a certain threshold, PVL levels in the herd tend to be lower. Furthermore, *BoLA-DRB3* polymorphism is known to be associated with PVL in milk [77]. Moreover, combining a biological barrier of resistant cattle in a stall-barn system with the preferential culling of susceptible cattle markedly reduces BLV prevalence and PVL, even when BLV-infected and uninfected animals are group-housed [58]. In free stalls, free barns, and grazing systems, PVL transmission risk can also be reduced by gradually increasing the proportion of resistant cattle in herds. Interestingly, cattle carrying the resistant allele BoLA-DRB3*014:01:01 were found in all three dairy farms examined in the present study. Thus, *BoLA-DRB3* polymorphism may be used in farm management to prevent BLV transmission in China. Although more farms need to be examined, this study suggests the potential for BLV eradication using resistant cattle in China’s Holstein population. In addition, more than 1000 cattle breeds exist worldwide, and most of their *BoLA-DRB3* diversity has not yet been investigated [91]. Therefore, to achieve BLV eradication across different farm systems, large-scale BLV surveys targeting Holstein farms as well as farms with other breeds in China and globally are necessary.

## Figures and Tables

**Table 1:** *BoLA-DRB3* allele frequencies in Holstein 289 cattle in China ^1^.

*BoLA-DRB3*	
Allele	Frequency (%)
*001:01 ^2^	32.70
*002:01	0.87
*003:01:01	0.17
*003:02:01	0.17
*004:01	0.17
*005:01	0.17
*005:03	0.35
*006:01	0.87
*007:01	0.87
*008:01	0.52
*009:01	0.69
*009:02	2.25
*010:01	5.88
*011:01	8.30
*011:02	0.35
*012:01	2.25
*014:01:01	17.30
*015:01	21.45
*016:01	0.35
*017:01	0.17
*018:01	0.87
*020:02:01	0.17
*027:01	0.17
*027:03	2.08
*031:01	0.35
*032:02	0.17
*044:01	0.17
*045:01	0.17

^1^ The most frequent alleles are underlined (greater than 5%). ^2^ *BoLA-DRB3* alleles were identified using PCR sequence-based typing.

**Table 2:** Detection of BLV provirus and anti-BLV antibodies by Dry Dual-CoCoMo assay and ELISA ^1^.

	H1		H2		H3		Total	
	Head	(%)	Head	(%)	Head	(%)	Head	(%)
Provirus(+)/Antibody(+)	28	31.5	18	18.0	8	8.0	54	18.7
Provirus(+)/Antibody(−)	0	0.0	2	2.0	2	2.0	4	1.4
Provirus(−)/Antibody(+)	0	0.0	1	1.0	0	0.0	1	0.3
Provirus(−)/Antibody(−)	61	68.5	79	79.0	90	90.0	230	79.6
Total	89		100		100		289	

^1^ A total of 289 blood samples were collected from three different farms (H1, H2, and H3) in Binzhou City, Shandong Province, China, and screened for BLV infection using a combination of enzyme-linked immunosorbent assay (ELISA) to detect the anti-BLV gp51 antibody and a real-time PCR test with Dry Dual-CoCoMo at the time of blood collection. Between the two methods for detecting BLV provirus and anti-BLV antibodies, at least one result was considered positive for BLV infection.

**Table 3:** Association of *BoLA-DRB3* alleles with BLV-infected and -non-infected Holstein cattle in China ^1^.

BoLA-DRB3 Alleles	Holstein				Fisher’s Exact Test		Susceptibility ^4^
	BLV-Infected Cattle n ^2^ = 59		BLV-Non-Infected Cattle n = 230		OR ^3^ (95%CI)	*p*-Value ^6^	
	Number of Alleles	Allele Frequency (%)	Number of Alleles	Allele Frequency (%)			
*001:01 ^7^	39	33.05	150	32.61	1.01 (0.84–1.25)	0.91	-
*002:01	0	0.00	5	1.09	0.00	0.59	-
*003:01:01	0	0.00	1	0.22	0.00	1.00	-
*003:02:01	0	0.00	1	0.22	0.00	1.00	-
*004:01	0	0.00	1	0.22	0.00	1.00	-
*005:01	1	0.85	0	0.00	Inf ^5^	0.20	-
*005:03	1	0.85	1	0.22	3.90 (0.07–220)	0.37	-
*006:01	0	0.00	5	1.09	0.00	0.59	-
*007:01	0	0.00	5	1.09	0.00	0.59	-
*008:01	0	0.00	3	0.65	0.00	1.00	-
*009:01	0	0.00	4	0.87	0.00	0.59	-
*009:02	0	0.00	13	2.83	0.00	0.08	-
*010:01	7	5.93	27	5.87	1.01 (0.64–1.61)	1.00	-
*011:01	17	14.41	31	6.74	2.14 (1.74–3.12)	0.01	**S**
*011:02	1	0.85	1	0.22	3.90 (0.07–220)	0.37	-
*012:01	1	0.85	12	2.61	0.32 (0.03–2.98)	0.48	-
*014:01:01	18	15.25	82	17.83	0.86 (0.64–1.07)	0.58	-
*015:01	28	23.73	96	20.87	1.14 (0.95–1.46)	0.53	-
*016:01	0	0.00	2	0.43	0.00	1.00	-
*017:01	1	0.85	0	0.00	Inf	0.20	-
*018:01	2	1.69	3	0.65	2.60 (0.46–15.0)	0.27	-
*020:02:01	1	0.85	0	0.00	Inf	0.20	-
*027:01	0	0.00	1	0.22	0.00	1.00	-
*027:03	1	0.85	11	2.39	0.35 (0.04–3.30)	0.48	-
*031:01	0	0.00	2	0.43	0.00	1.00	-
*032:02	0	0.00	1	0.22	0.00	1.00	-
*044:01	0	0.00	1	0.22	0.00	1.00	-
*045:01	0	0.00	1	0.22	0.00	1.00	-

^1^ Association between *BoLA-DRB3* allele and BLV infection was determined based on Fisher’s exact test by comparing the frequency distribution of alleles between BLV-infected and non-infected Holstein cattle. ^2^ n, total number of cattle; ^3^ OR, odds ratio; ^4^ Susceptibility, S = Susceptibility, ^5^ Inf, Infinity. ^6^ *p*-values < 0.05 and <0.05/28 were considered statistically significant and strictly statistically significant, respectively. ^7^ *BoLA-DRB3* alleles were identified using PCR sequence-based typing.

**Table 4:** The mean and range of proviral load (PVL) of BLV infected cattle from three farms.

Farm Number	Number of Cattle (Head)	PVL	
		Mean (Copies/10^5^ Cells)	Range (Copies/10^5^ Cells)
H1	89	43,722	8–128,378
H2	100	25,479	0–82,097
H3	100	21,988	9–79,832

**Table 5:** Summary of CoCoMo-qPCR-based proviral load (PVL) and gp51 antibodies determination for the BLV and classification criteria for PVL.

Number of Tested Animals (Heads)	Number of Positive Samples (Heads)	Mean PVL (Copies/10^5^ Cells)	PVL Category	PVL Range (Copies/10^5^ Cells)	Number of Cattle (Head)
289	59	33,569	Low PVL (LPVL)	0–3606	19
			Moderate	3720–50,743	20
			High PVL (HPVL)	51,531–128,378	20

**Table 6:** Association between *BoLA-DRB3* alleles and BLV proviral load (PVL) in Holstein cattle in China ^1^.

*BoLA-DRB3* Alleles	Holstein				Fisher’s Exact Test		Susceptibility ^5^
	LPVL ^2^ (n ^3^ = 19)		HPVL ^2^ (n = 20)		OR ^4^ (95%CI)	*p*-Value ^8^	
	Number of Alleles	Allele Frequency (%)	Number of Alleles	Allele Frequency (%)			
*001:01 ^9^	15	39.5	14	35.0	1.13 (0.58–2.54)	0.81	-
*005:01	0	0.0	1	2.5	0.00	1.00	-
*010:01	0	0.0	4	10.0	0.00	0.12	-
*011:01	7	18.4	8	20.0	0.92 (0.35–2.30)	1.00	-
*011:02	1	2.6	0	0.0	Inf ^6^	0.48	-
*012:01	0	0.0	1	2.5	0.00	1.00	-
*014:01:01	9	23.7	0	0.0	Inf	0.001 ^7^	R
*015:01	5	13.2	10	25.0	0.53 (0.167–1.24)	0.25	-
*018:01	0	0.0	2	5.0	0	0.49	-
*020:02:01	1	2.6	0	0.0	Inf	0.48	-

^1^ Association between *BoLA-DRB3* allele and PVL profile was determined based on Fisher’s exact test by comparing the frequency distribution of alleles between LPVL and HPVL cattle. ^2^ HPVL: high proviral load; LPVL: low proviral load; ^3^ n, total cattle number; ^4^ OR, odds ratio; ^5^ Susceptibility, R = resistance, ^6^ Inf, Infinity. ^7^ Statistical power was 0.86 and was considered adequate. ^8^ *p*-values < 0.05 and <0.05/10 were considered statistically significant and strictly statistically significant, respectively. ^9^ *BoLA-DRB3* alleles were identified using PCR sequence-based typing.

**Table 7:** *BoLA-DRB3* diversity in Holstein cattle.

Country	Cattle No.	Heard Region	Number of Different *BoLA-DRB3* Allele	Major Alleles (Frequencies > 10%)	Reference
Chinese	289	3	28	*001:01, *015:01, *014:01:01	[this study]
Egyptian	121	3	18	*015:01, *011:01, *001:01	[73]
Vietnamese	81	-	27	*001:01, *012:01, *015:01, *027:03	[72]
Argentinean	424	4	33	*001:01, *015:01, *011:01	[83]
Bolivian	159	2	23	*015:01, *009:02, *011:01, *010:01, *006:01	[83]
Chilean	113	4	21	*015:01, *001:01, *011:01	[83]
Japanese	433	-	32	*015:01, *011:01, *001:01	[54]
	102	-	18	*011:01, *012:01, *015:01, *001:01	[81]
Paraguayan	127	5	26	*001:01, *015:01, *011:01	[83]
Peruvian	133	2	20	*015:01, *011:01, *001:01	[83]

## Data Availability

The data presented in this study are available upon request from the corresponding author.
